# Advancing PROMs for health system use in Canada and beyond

**DOI:** 10.1186/s41687-021-00370-6

**Published:** 2021-10-12

**Authors:** Michael Terner, Krista Louie, Candy Chow, Greg Webster

**Affiliations:** Canadian Institute for Health Information, 4110 Yonge Street, Suite 300, Toronto, ON M2P 2B7 Canada

**Keywords:** PROMs, Canada, Health system reporting, Data standards, Hip and knee arthroplasty

## Abstract

PROMs are essential to delivering patient-centred health care, and when applied routinely they can enhance communication between patients and providers, inform decisions for value-based health system improvements and improve overall patient care experiences and outcomes. The use of patient-reported outcome measures (PROMs) across Canada varies across provinces and territories, partly because of differences in health care delivery models across these jurisdictions. A national program that coordinates uses of PROMs is needed to ensure that this information is comparable across jurisdictions. This commentary provides a summary look at the development of national PROMs data standards and reporting for hip and knee replacement surgery, including the selection of survey tools, building consensus, developing and promoting standards, and reporting on the results nationally and internationally as well as outlining recent learnings from regional implementation of data standards. In 2017, the Canadian Institute for Health Information published national PROMs data collection standards for hip and knee arthroplasty that included guidelines for survey time points, the minimum data set and PROMs instruments. This broad-scale PROMs collection initiative had stakeholder engagement and support from multiple levels within the health system, including administrators, clinic managers, patients, and health system decision-makers. Learnings from regional implementation of the standards demonstrated the importance of assessing existing infrastructure and information technology requirements, mapping clinical workflows, planning for human and information technology resources, navigating local legislation and hospital policies and ensuring data linkage capabilities. This initiative showed the need for a common regional approach for PROMs collection to be efficient and effective. The learnings from implementation of the national Canadian PROMs program for hip and knee arthroplasty can be used as an example for other jurisdictions and clinical areas such as renal care and mental health. Common data standards allow for secondary use of this data that is valuable for reporting and informing policy and guidelines as well as meeting care delivery goals to further the shift in health care systems becoming more patient-centred to improve the quality-of-life of patients.

## Introduction

As Canada shifts to more patient-centred health care systems, input and feedback from patients is becoming increasingly valuable to achieve this shift. For health system use, patient-reported outcome measures (PROMs) are used to identify patients who could benefit from interventions, as a decision tool for clinical care pathways, as a platform for discussion around patient expectations, and for providing direct feedback on the effectivess of care [1]. The use of PROMs across Canada varies across jurisdictions and therefore Canada needs coordination from a national program that standardizes routine administration of PROMs for use in health services management, quality improvement and performance measurement. Given the range of possible uses of PROMs information, there are significant benefits for Canada that can be achieved through a coordinated approach to PROMs data collection to make this information available to clinicians, health system administrators, policy-makers, researchers and patients [1, 2].

The Canadian Institute for Health Information (CIHI) is an independent, pan-Canadian organization that provides health system databases, measurements and standards, and reports and analyses for decision-making at the local, regional and national levels. As such, CIHI is well positioned to coordinate standards, collection and reporting of PROMs data across the country. This commentary’s objectives are to provide a summary look at the development of national PROMs data standards and reporting that is still an emerging field in Canada and recent learnings from regional implementation of a data standard for PROMs before and after hip and knee replacement surgery.

## PROMs collaborations and developing data standards

In 2014, CIHI and Statistics Canada co-hosted a consensus conference to identify priority areas for enhanced PROMs information and subsequently established the PROMs forum to provide an opportunity for Canadian health leaders to discuss PROMs and to explore opportunities for advancing a common approach to PROMs in Canada [3]. Next steps from the forum included advancing more collaborative project work, including the establishment of the PROMs Advisory Committee [3]. Within this committee, clinical experts in hip and knee replacements and renal care highlighted opportunities in these areas where a demonstration project could illustrate the value of PROMs to enhance patient care and outcomes.

With stakeholder support, the PROMs Hip and Knee Replacement Working Group was formed to guide the development of a PROMs data standard and reporting in hip and knee arthroplasty across Canada. Members included representatives from ministries of health, orthopaedic surgeons, and researchers in six jurisdictions actively involved with PROMs data collection. The group conducted a literature review of over 96 arcticles and an environmental scan of 21 joint replacement programs and registries in Canada and internationally, reviewed input on recommended generic tools from the International Consortium of Health Outcomes Measurement (ICHOM) and the International Society of Arthroplasty Registries (ISAR) PROMs Working Group on the appropriateness of tools for patient who have arthroplasty [4, 5], consulted and engaged members of the PROMs Hip and Knee Replacement Working Group and the Canadian Joint Replacement Registry to develop the standards. The assessment of the instruments included psychometric properties (e.g., reliability and validity), clinical and health system applicability, patient engagement during development, collection burden, translations and validations available, licensing and costs, and current use [2, 6].

A short-list of commonly used PROMs in routine care for hip and knee arthroplasty was circulated to the PROMs Hip and Knee Replacement Working Group. In 2017, this Working Group and the Canadian Joint Replacement Registry Advisory Committee both approved and endorsed the national PROMs data collection standards for hip and knee arthroplasty and CIHI published the standards [7]. The national PROMs standards include guidelines for survey time points, the minimum data set and PROMs instruments [7]. The standards include the Canadian English and French versions of the the Oxford Hip Score (OHS), the Oxford Knee Score (OKS) and the EQ-5D-5L, administered concurrently with a patient experience question and an item on overall satisfaction with the treatment outcome [7]. The EQ-5D-5L is a generic tool that has been translated and validated in over 130 languages and is free for non-commercial use [8]. It is a generic tool with the capability to facilitate comparisons of cost-effectiveness based on calculated Quality-Adjusted Life Years and enables international comparisons [4]. The OHS/OKS are short instruments that allows for overall summary score as well as pain and function sub-scores[9, 10], areas of focus for hip and knee arthroplasty. Furthermore, these instruments area used in existing routine care programs across Canada which maxmizes participation [3].

## Learnings from Implementation of the standards

Canada's publicly funded health care system is primarily the responsibility of the provinces and territories with funding and coordination shared between federal, provincial/territorial and municipal governments. When developing a pan-Canadian program for PROMs, significant consideration was given to the separate jurisdictional responsibilities, including differences in health care delivery models (e.g., remote locations, decentralized services) and cultural context (e.g., indigenous populations). As a national health care organization, CIHI was able to release and recommend the national standards for adoption while ensuring that PROMs data is collected in compliance with privacy regulations. To meet the purpose and needs of different jurisdications, the PROMs Hip and Knee Replacement Working Group incorporated regional representation during consultations for the standard. Ultimately, a major incentive for adoption was to enable pan-Canadian and international comparisons and reporting.

This broad-scale PROMs collection initiative had stakeholder engagement and support from multiple levels within the health system [7], including administrators, clinic managers, patients, and health system decision-makers [3]. Support from clinical champions led to buy-in from surgeons, care teams and patients, and promoted the value of PROMs in routine care [11]. The absence of involvement from these key stakeholders may lead to lack of understanding of the intended purpose, underdeveloped infrastructure and processes for collection, and results in lower response rates and less robust data [11].

This initiative showed the need for a common regional approach for PROMs collection to be efficient and effective. The following learnings describe the need for adequate planning, resources and deployment during implementation.

- Assessment of existing infrastructure for data collection is imperative in order to determine the need for new or enhanced infrastructure or supports. In Ontario, the agency responsible for coordinating data collection leveraged the existing Cancer PROMs e-platform, already established in hospitals, and adapted the infrastructure for patients having hip and knee arthroplasty.
- Mapping of clinical workflows for data collection and data flow is needed during the planning phase to ensure optimal efficiency of data collection. For collection in Ontario, consultation with specific care teams confirmed existing research programs could be leveraged.
- It is important to consider information technology requirements for integration and interoperability to reduce patient and provider burden [12]. For example availability of PROMs data into electronic health records made these data readily accessible to patients, improving patient–provider communication and informing medical decision-making [13].
- Assessing and planning human and information technology resources for ongoing collection and follow-up is vital for a sustainable program.

Planning for longitudinal data is particularly important for hip and knee arthroplasty PROMs that require administration before and after surgery to measure the post-operative change. Following-up for the post-operative time point does not have to be resource intensive. For example, automated electronic notifications from a known entity—such as a health care provider—are efficient in increasing response rate. The use of technology for these notications, such as mobile applications, automated telephone calls, email reminders have helped increase response rates. Supports can also come in the form of shared learnings and experiences such as how to successfully navigate local legislation or hospital policies around using emails to facilitate PROMs collection. As shown during the COVID-19 pandemic with transitions to virtual care, patient care and the associated pathways are dynamic and consequently data collection needs to be equally dynamic. For example, infrastructure is needed that allows for flexibility in how information is collected to fit the needs of patients and providers [11].

For optimal usability of PROMs data, planning to ensure data linkage capabilities (including a common identifier and relevant privacy policies) is essential to reduce redundant collect of data that are available in clinical and administrative systems. Access to clinical and administrative data systems also allows for case-mix adjustments based on demographic and clinical factors, which are recommended for obtaining meaningful comparisons across jurisdictions and care providers.

## Using PROMs data for regional, national and international reporting

An extensive amount of planning and resources are dedicated to the implementation and ongoing collection of PROMs for hip and knee replacments and an equal amount of research and development needs to be focused on the reporting and use of these data such that it reflects the purpose data collection. Health outcomes information can be collected at various levels for a range of different purposes, from clinical to policy-making [14]. Beyond primary uses of PROMs at the point of care, secondary use of this data is valuable for reporting and informing policy and guidelines as well as meeting care delivery goals [14]. Alberta has been broadly collecting and using PROMs by incorporating the EQ-5D in population health surveys since 2010. Within the province, PROMs are reported in various programs including balanced scorecards to support continuous improvement programs [15]. Ontario is using PROMs data from patients receiving hip or knee arthroplasty to meet the quadruple aim and ensuring delivery of value-based care [16]. In addition, reporting at the health system level is used to compare outcomes across health systems (including at the facility, regional and national/international levels) to identify best practices and drive quality improvement. During the development of indicators and reporting, input from stakeholders is imperative to ensure they are relevant and actionable for clinical use and health system evaluation [17].

The OECD releases the bi-annual *Health at a Glance* publication that includes internationally comparable indicators to support health system performance. In 2017, the OECD Health Committee launched the Patient-Reported Indicator Surveys (PaRIS) initiative to build international capacity to measure and compare patient-reported indicators [18]. The PaRIS Working Group for Hip and Knee Replacement Surgery, with representation from the international community, agreed on indicators for international reporting. Standardized by age, sex and pre-operative score, these indicators for hip and knee replacement were:

- Adjusted mean change between pre- and post-operative EQ-5D-3L scores
- Adjusted mean change between pre- and post-operative OHS and HOOS-PS scores
- Adjusted mean change between pre- and post-operative OKS and KOOS-PS scores
- Quality-adjusted life years

Comorbidities were not included for risk-adjustment because of challenges collecting this information consistently at the time of the first publication of the indicators. International comparisons for these indicators are currently limited to registries and programs collecting the same PROMs instrument or instruments with an available crosswalk. Ongoing alignment and increased adoption of the international hip and knee replacement standards has the potential to make international PROMs data more directly comparable and robust.

## Future direction

CIHI continues to support the advancement of PROMs in Canada and internationally through promotion and implementation of the hip and knee replacement standards and and evaluating the feasibility of collecting PROMs data through existing administrative data channels. The 2019 OECD’s Health at a Glance report included for the first time PROMs indicators for hip and knee arthroplasty and breast cancer [19]. For the 2021 release of this report, the working groups within the PaRIS initiative are working to expand participation in hip and knee arthroplasty and breast cancer to more international partner countries as well as including mental health PROMs.

Establishing a PROMs-focused program at CIHI that subsequently developed and promoted standards for PROMs data collection and reporting for hip and knee arthroplasty has important lessons for further work in increasing adoption of common standards in other clinical areas. Other clinical areas that have been identified for expansion of generic and condition-specific instruments include renal care and mental health. Experience with implementing and promoting standards across jurisdictions showed the need for a recommended procedure to avoid challenges that occur in promoting a voluntary standard. PROMs data can benefit both the patient and provider as well as inform policy and best practices through both primary and secondary uses, including providing comparable effectiveness of outcomes that can only be measured through patient reporting. PROMs can complement clinical outcomes and indicators for facility-, provincial-, and international-level reporting to shift health care systems to be more patient-centred with value-based procedures that improve the quality-of-life of Canadians and beyond.

## Data Availability

Not applicable.

## Abbreviations

PROMs: Patient-reported outcome measures
CIHI: Canadian Institute for Health Information
ICHOM: International Consortium of Health Outcomes Measurement
ISAR: International Society of Arthroplasty Registries
OHS: Oxford Hip Score
OKS: Oxford Knee Score
OECD: Organisation for Economic Co-operation and Development
PaRIS: Patient-Reported Indicator Surveys
HOOS-PS scores: Hip disability and Osteoarthritis Outcome Score Physical Function Short form
KOOS-PS scores: Knee disability and Osteoarthritis Outcome Score Physical Function Short form
